# The American Public Health Association—Second Annual Session

**Published:** 1874-12-15

**Authors:** 


### THE AMERICAN PUBLIC HEALTH ASSOCIATION—SECOND ANNUAL SESSION.

*From The Philadelphia Medical and Surgical Reporter.*

THE second annual session of the American Public Health Association commenced November 11th, at 12 o'clock, in the hall of the College of Physicians, this city.

The President, Dr. Stephen Smith, made a few introductory remarks relative to the progress of the work of the association during the past year.

Prof. Henry Hartshorne then made an address of welcome, after which he read a paper on "Excessive Infant Mortality of Cities and the Means of its Prevention."

J. R. Black, M.D., of Ohio, next read a paper on "The Influence of Hereditary Defects upon the Health of the People, with suggestions in regard to Prevention and Eradication." From this paper we make the following abstract:—

He said that the doctrine may now be said to be established that organization and function are one, or that there is in the body no independent spirit or principle apart from that inherent in the various forms of organized matter. The popular exclusion of this doctrine in questions of hygiene and of ethics, or the trust reposed in addressing every reformatory effort of this kind to an abstract ethereal entity either in or above ourselves, is justly chargeable with the terrible indictment of being the main influence by which mankind have been made the most pain-stricken, the most sickly, the most frequently and fearfully deformed, and the most likely to die at an untimely period, and as malefactors in torment, and for the vindication of law, of all other animated beings.

It has caused worthy persons to believe that afflictions and sickness are sent, not brought upon ourselves, and that while it is a binding duty to care for the sick, the deaf, the maimed, the idiotic and the insane, it is scarcely thought to be obligatory in a public or personal sense to prevent, through purely mundane instrumentalities, any or all of these evils from ever afflicting our race.

A hereditary defect may imply a disease directly transmitted, as in syphilis or scrofula; or a deformity, as in harelip, or simply tendency to some disease, as in insanity or tuberculosis. The way in which ordinary forms of hereditary defects originate is not difficult to comprehend. It is often practically demonstrated to every competent observer, especially in the large cities.

As a rule, the residents of a salubrious country district are freer from taints of blood and defects of organization than those of a city, and a removal of persons into the latter place produces an impairment in health of a transmissible quality. The digestive organs are the first of the vital harmonies to fail from bad habits of life.

If those habits be continued for a generation or two an inbred weakness of these organs will become an inheritance of the offspring. If the infringement of vital law consists of great mental strain, or in the continued and excessive use of stimulants and narcotics, some form of nervous impairment will ensue, which, if prolonged, may end in insanity or predisposition to attacks of nervous disorder.

If the syphilitic taint is engrafted upon the blood, this, with insufficient out-door exercise, and the long-continued breathing of impure house air, will be sure to give rise to pulmonary consumption.

Those who have given the laws of health any attention are aware that there are few persons who do not violate them, nor is obedience at all impracticable.

Of late the achievements of science have actually tended to produce an increase in the number of degenerate men and women, because every one does not know and act for himself in sanitary matters, but relies on the knowledge confined to a few scientists. The latter cannot manipulate health, vigor, and good constitutions into their fellow-beings.

The first and great requisite to prevent all this is knowledge of what constitutes true vigor, sympathy and health. Not a very few persons are of the opinion that these conditions are very well known to the popular mind. Observation has led to a very different conclusion. Many have gained vague ideas on the subject most frequently from those who have more vanity. A few thoroughly understand the purpose of one or more of the conditions of health, and, perhaps, attach an undue importance to them. This knowledge, to be useful, needs to be personal and thorough; no mere elementary smattering to which the mind may passively assent, but such a deep and thorough familiarity with the subject which will enforce the conviction that the alternatives of pleasure or pain, health or sickness, long lives or short ones, are, except from chances infinitesimally small, wholly in our power. Precisely that which prevents sickness will also prevent the stamping of an inherent defect upon the organization.

Dr. Richardson, of this city, at the conclusion of Dr. Black's paper, made a few remarks on the subject of hereditary disease, which he argued was the effect of a law of nature, the opposite of the "survival of the fittest," and which he had formulated three years ago as the "extinction of the unfit."

Dr. Samuel Osgood, of New York, spoke of the hereditary tendencies in the disposition of children which he had noticed in his pastoral duties. He had asked whether hereditary tendencies were easier to check than those of a personal origin, and was glad to hear that they were. As to the survival of the fittest, he contended that that was not always the case. The worst men and brutes frequently outlived the best of their fellows.

A paper on "The Health of the Tenement Populations and the Sanitary requirements of their Dwellings," by Edward H. Janes, M.D., of New York, was next read by the secretary, as Dr. Janes was not present.

The next paper was a report upon the death-rate of each sex in Michigan, and comparison with "Dr. Farr's Life Tables of Healthy Districts of England," by H. B. Baker, M.D., secretary of the State Board of Health.

Dr. J. S. Billings presented a paper on hospital location and construction, from which we make the following extract:—

Experience has shown that large and costly hospitals, even on the pavilion plan, are not necessarily free from the evils indicated by the word "hospitalism;" and practical trial, in our late war, repeated and confirmed more recently in Europe, has led to the recommendation that hospitals should be temporary wooden structures, intended to last but ten or twelve years. The good results obtained in our large military hospitals were not alone due to their temporary character, for the morbific element due to length of occupation did not have time to develop in them. They were better located than civil hospitals, being in the country, where there was plenty of room and fresh air. The class of patients was better, the control over them more efficient, and they were more readily classified than in civil life, thus lessening the evil (to which I shall presently refer) of placing a number of men in one room, with different diseases and wants.

When cases of zymotic disease occurred, tents were largely used, and the more they were employed the better the result. In an economical point of view it is evident that if one-half the money required for brick or stone structures was used to erect plain balloon-frame wooden buildings, and the other half invested at ordinary rate of interest, at the end of about twelve years the amount on hand would be what it was in the beginning, the old buildings could be removed, and the process repeated, thus giving a new hospital every twelve years. The necessary buildings for the care of two hundred patients should be constructed for about $50,000. The smaller the number of patients the greater the cost per bed. Thus a hospital for one hundred patients will cost about $35,00, for fifty patients $12,000, etc. Our large metropolitan hospitals usually are, and should be, connected with medical schools, and, on account of accessibility, it is generally considered necessary to place them in or very near the city, where space is limited and costly. Dr. Billings doubts very much whether this supposed necessity exists, and whether it would not be possible to place hospitals five or ten miles away from the city, where they could have ample space, and place the medical colleges with them.

The paper also states many special advantages which pertain to floating hospitals, a class of structure of which more use could be made. It is suggested that a floating hospital might be constructed on flat-bottomed boats, radiating from a central triangle or polygon. Such a hospital could most conveniently be arranged for three hundred beds or less, and where space can be more conveniently obtained on water than on land would serve an excellent purpose, but the temporary character of the structure must be insisted on.

Two valuable papers, one on the "Sanitary Relations of Hospitals," by William Pepper, M.D., the other on "Hospital Architecture, and the Perfect Ventilation of Hospital Wards," by Carl Pfeiffer, of New York, were next read, and, together with all the preceding papers, referred to the Committee on Publication. The last hour of the afternoon session was passed in a conference of sanitary officers and others upon methods and experience in the public health service.

EVENING SESSION.

The evening session opened at half-past seven o'clock with a large attendance. A fair proportion of the audience were ladies. Hon. Morton Mc Michael presided, and introduced the exercises of the evening with an address. At the conclusion of the honorable gentleman's address, Rev. Samuel Osgood, D.D., was introduced, and delivered a discourse on "The Relations of Health and Higher Culture."

The speaker, after noting the difference between modern society and the society of the ancients, said that the demands upon us had increased until we were in danger, as a race, of becoming nervous, sickly and discontented. Health, he continued, was a part of higher culture, for without a sound body there could not be a sound mind. If life was the continuous adjustment of outward and inner relations, then health could only be obtained by a patient study and religious following of nature's laws. Whatever might be the aspirations of the soul, our knowledge must come through the senses, but unless the senses were perfect in action and thoroughly trained, the mind could not be advanced. Reference was then made to the bad methods of cooking food in this country. He said our vices and follies come in great part from what goes into the mouth. The cannon and the sword had at times done terrible work, but the pipe and the bottle, the cigar box and the whisky cask, were likely to beat them both.

Prof. S. D. Gross, M.D., then read an elaborate discourse upon "The Factors of Disease and Death after Injuries, Parturition, and Surgical Operations," a paper on hospitals in their relations to public health interests, and the economy of perfect care of the sick and hurt. In his treatment of the subjects, Dr. Gross particularly dwelt upon the necessity of employing the most scrupulous neatness in all surgical operations. In speaking of the effects of bad drainage, mention was made of the appalling epidemics which raged for a time in a ladies' school at Pittsfield, Mass., and later at a hotel in Washington. The poisons of infectious and cutaneous diseases were next treated. The speaker related many instances in which the poisons of various diseases were communicated from person to person in an almost unaccountable manner. The specific poisons of cholera, diphtheria, small-pox, scarlatina, were referred to as particularly tenacious and potent. The average mortality from zymotic diseases was 26½ per cent. of all deaths.

In treating of hospitals the Doctor said very plainly that the mortality in most of them was frightful. The Episcopal Hospital and the Hospital of the University of Pennsylvania were referred to as well planned. He said, however, that no single ward should have more than six or eight beds, and no hospital should accommodate more than one hundred patients. The Doctor referred in warm terms to the necessity of erecting convalescent hospitals, where patients who had passed the crisis of disease could recover their strength without danger of infection from persons afflicted with other diseases. The discourse concluded with a graphic description of the condition of the tenement districts of great cities and the means whereby they might be renovated.

On motion, a vote of thanks was unanimously tendered to Rev. Samuel Osgood, D.D., and Dr. Gross, for the interest they had afforded.

The association then adjourned.

### SECOND DAY.

The association convened at nine o'clock. President Stephen Smith, M.D., occupied the chair; Dr. E. Harris, secretary. The first paper presented was on the subject of "Building Ground in its Relation to Health and Disease," by Ezra M. Hunt, M.D., President of the Sanitary Commission of New Jersey. It states that the condition of the ground has very much to do with all questions of health. The character of the soil, the degree to which it can dispose of all that comes in contact with it, whether in the form of gases of animal or vegetable decay, or of pure and impure liquids, all have intrinsic and vital bearings upon human health.

Where natural transformations are in no wise interfered with by art it is wonderful to see how processes involving productions inimical to health are so conducted as to be entirely consistent with vigorous existence. While decomposition is the rule, evil therefrom under natural conditions is the exception. While, for instance, enough carbonic acid is produced each day to kill all the inhabitants of the earth, yet it is so well managed as not to interfere with the health of man as animal. But the very moment a spot comes to be builded upon it is by necessity placed in abnormal conditions.

The building clears the ground of that herbage which had no unimportant sanitary office in appropriating the products of decay. It covers it from sunlight and sun heat, and necessarily makes its condition as to these quite different. It interferes with the range of the winds, and modifies the immediate thermometric and hygrometric condition of the atmosphere. It throws the rain-fall into streams upon the ground around its sides, rather than allowing it to diffuse itself as it does in drops.

It is believed that one of the causes of the prevalence of such fevers as typhus and typhoid, in the winter, is that the greater inner heat of houses causes the currents of air from the surrounding ground to set to them, under the general law of currents as affected by heat. If the soil air is polluted by sewerage or only by the interruption of those processes which Nature has instituted for purifying it, we are sharers in that contaminated air.

The fact of water in the ground is more apparent than that of air, but still its relations thereto are underrated in its sanitary bearings. There is a depth varying with the soil and locality at which the ground water is in general intended to fill up the space between earth particles. But in several feet of the ground nearest to the surface it is intended that the soil should have both air and water in circulation. Between them and heat there is a correlation and conservation which is conducted as wonderfully and as scientifically below ground as above it.

This condition, when uninterrupted, tends healthward, but when suspended contaminates the ground. The capacity of the ground for air is already shown, and by expelling the air from dried earth, or, in other words, by pouring into it water, we find its capacity for water. Such grounds as we are familiar with will thus take in fifty per cent. in volume of water, and even most marble will hold four per cent. The paper further states that in cities we need more dry-earth system. Perfect under drainage is the first great need of most cities. Regulations of cellars, and of all other holes below the surface is the next great study.

We must get the homes of the people on a better foundation than damp, water-soaked, air-polluted, filth-burdened ground.

Remarks on Dr. Hunt's paper were made by Professor Henry Hartshorne, of this city; Dr. John H. Rauch, of Illinois; Dr. Ray, of this city; Dr. Bell of New York, and Dr. John A. Stewart, of Baltimore. A motion made by Dr. Hartshorne that the paper should be referred to the Publication Committee, was carried.

Dr. S. C. Busey, of Washington, D. C., presented a report upon the gathering, packing, transportation, and sale of fresh vegetables and fruits, and the competent inspection and free markets for producers of the same.

### AFTERNOON SESSION.

At the afternoon session Dr. Edwin M. Snow, Superintendent of Health, Providence, Rhode Island, occupied the chair. The first paper, by Dr. E. Harris, of New York, "A report upon the vital statistics, and the methods of public health administration in the cities and large towns of North America," was not read on account of its extreme length, but was referred to the committee for publication. Dr. Joseph M. Toner of Washington, D. C., then read an elaborate treatise on "Conditions and accidents which endanger, limit, or prevent vaccination from giving full protection from small-pox."

From this paper we make the following abstract:

Vaccinators in Great Britain are required to stand an examination as to their qualifications before receiving an appointment. I apprehend that great benefit would accrue to the people of the United States if the public vaccinators were appointed by state and city governments. I but assert the conviction of not only every medical man, but of every intelligent citizen, that a properly performed and successful vaccination, whether with humanized or animal virus, is as complete a protection against small-pox now as it ever was, and is a more perfect prophylactic than we possess against any other known disease.

*Spurious Vaccination.*—This general head may comprise all we have to say on deviations in the character of vaccine virus, and deviations from the normal course of the true protective vesicle. Perfectly good vaccine lymph, even in the primary vaccination, may produce a spurious pustule, and consequently secure no immunity from small-pox, and it is the duty of the vaccinator to remedy and detect this accident. If the papular state be hastened the vesicle will be illy formed, and the lymph opaque and unfit to use in propagating the disease, and does not promise complete protection. The centre of the vesicle in such a case is not well defined, and the regular stages of the early development have been interrupted, and the areolar either does not form or is not of normal appearance. A condition must always be suspicious in the development of any undue itching set up about the second or third day. Where the papulæ assume a conoidal shape about the fifth day, and have a straw-colored or opaque lymph, or broken, ragged, weeping vesicle, with an ill-defined areolar about the sixth or seventh day, it can at once be pronounced as spurious. Vaccination may be retarded somewhat in its course, but I think it can never be accelerated beyond a day or so without destroying its protective character.

When the reading of this paper was concluded, Dr. Moreau Morris, of New York, said that the first point seemed to be how to get vaccine virus. So far as his experience extended he was satisfied that humanized lymph was equally protective with bovine. Concerning the collection of virus, its use, and its introduction, he referred to the mode of collecting virus used by physicians, and said that he believed that vaccine should be kept at an even temperature, and not preserved beyond a certain length of time. Physicians should be assured that the system had been thoroughly saturated with the virus before regarding the vaccination as protective.

Dr. Snow stated that out of five hundred children vaccinated by him only one had afterwards taken the small-pox.

Dr. Toner's paper was, on motion, referred to the Committee of Publication, after which Dr. Edwin M. Snow read a paper on the question: "Does Small-Pox become Epidemic?" After reviewing the apparent epidemics of small-pox which had visited American and European cities, from time to time, the Doctor said: It seems that we may safely conclude that the small-pox of the winter of 1872–73 did not possess the important characteristic of a true epidemic, of being widespread over the country at the same time. We understand by an epidemic influence some cause of disease which is widespread in its effect upon the people, which is independent of the ordinary or sporadic cause or causes, and which in itself and by itself has some power toward producing disease. Take for illustration: When Asiatic cholera is truly epidemic there is widespread over the country an influence which, of itself, tends to produce cholera, and which, in connection with local causes, does produce it, and without which the cholera cannot exist, even though all the local causes may be present. Can we conceive of any influence that can be directly called, in connection with small-pox, epidemic? One hundred cases of small-pox occur at the present day without contagion, either direct or indirect. My conclusion is that the great prevalence of this disease in Philadelphia in the year 1871, and in other cities, from time to time, had no connection with any true epidemic influence, but was due solely to the great number of cases of disease existing at the same time in a crowded city.

The next paper was on the "Causation of Scarlatina, with reference to the contagious and epidemic attributes, as illustrated in the course of that disease in the twenty-fourth ward of New York." Dr. C. F. Rodenstein, the author, stated that there would not be time to read the treatise. He then explained that by a series of experiments he had discovered in one locality that the disease was spread almost entirely by drinking water.

Benj. C. Miller, M.D., Sanitary Superintendent of Chicago, then briefly sketched his paper on the "Methods of Treatment of Gases from Rendering Tanks, and the Disposal of Tank Offal." He stated that if the progress in the future was equal to that which had been made in the past, there could be little cause for complaint against the packing-houses of Chicago. Adjourned.

### THIRD DAY.

The association re-assembled at nine o'clock. President Stephen Smith,

M.D., occupied the chair. Dr. H. B. Baker, secretary of the Board of Health of Michigan, presented the association a portfolio containing specimens of poisonous wall-paper collected in different parts of the state. Prof. J. LeConte, of this city, said he was glad the subject had been opened, and called attention to the indiscriminate use of poisonous substances in agriculture. He thought the matter should be referred to a scientific commission.

Dr. John M. Woodworth said that every man of science in the United States viewed with horror the extent of this abuse. He moved that the Executive Committee should be requested to consider the propriety of appointing a special committee to confer and report on the subject. This was carried unanimously.

A communication from Dr. Francis Bacon, of Yale College Medical School, inviting the association to hold its next annual meeting at New Haven, Conn., was laid on the table.

It was announced that the subject of slaughter houses in large cities would be discussed some time this morning.

Stephen Smith, M.D., of New York, then read a paper on "The Reciprocal Relations of the Public Health Service and the Highest Educational Qualifications of the Medical Profession." From this we make the following abstract:

This review of the state of the medical art during the early periods of Roman history conveys a suggestive and useful lesson. If we were to search our statistics for evidences of the rank and position of the medical profession, as we search the Justinian code for substantial proofs of the position of the medical profession at Rome in different periods of history, we would find the highest conception of a physician to which American law had attained was defined by competent legal authority as follows: "The term 'physician' may be applied to any one who publicly announces himself to be a practitioner of this art, and undertakes to treat the sick either for or without reward" (Ordronaux). We might very justly infer from this definition that medicine as a science and an art was unknown in this country, and that medical practice was placed on the same plane as the most common trade, and our conclusions from these data would not only be logically correct but they would be historically true. Before the law medicine has occupied the position of the most ordinary handicraft, and has been subject to the same legal restrictions and obligations. While the historian who consulted only our statute books might reasonably conclude that scientific medicine had no recognition and hence no existence in the United States for one hundred years, our literature and our institutions would give ample evidence of not only the existence of medical science and medical art, but of its activity. A more rational conclusion to which the philosophical historian would come would be that scientific medicine secured and maintained whatever rank it held by its own unaided efforts.

After expressing a hope that in future the term physician might be better defined than in the past, the Doctor continued: It requires but little penetration to discover that there is a growing confidence in American communities in preventive medicine. Public health service can never inspire the proper degree of confidence unless it is sustained by medical science and medical art, in their highest degree of development. This science requires an organization with every needed scientific appointment, which shall be capable of searching out all the hidden sources of disease, and be of service in warding off pestilence, or mitigating its severity. It will also seek out and correct all those conditions which tend to deteriorate the physical condition of each generation, which impair development and which diminish longevity. Its real efficiency and success must depend primarily upon the state of development of the medical sciences, the extent to which such service relies upon these sciences, and in their application in practice. The relations between the two, health source and the development of scientific and practical medicine, were assumed by the writer to be reciprocal, inasmuch as they were so intimately related that it was impossible for the former to advance without a corresponding advance of the latter. The paper continued upon an elaboration of this statement, finally closing with a hope that the Centennial of American Freedom should also see the Centennial of Public Health Service, and mark its close union with an advanced medical profession.

Dr. Frederick R. Sturgis, M.D., of New York, followed with an exhaustive paper upon "The Relation of Syphilis to the Public Health," after which Dr. George M. Beard, of New York, presented a paper on "Hay Fever, or Summer Catarrh." This paper shows that, from facts which Dr. Beard has gathered, he is obliged to make deductions diametrically opposed to all existing theories respecting hay fever. He regards it a complex, and not a simple disease. The first factor is a nervous temperament. The second is heat following cold. The third factor is some exciting cause, as dust, cinders, hay (fresh mown), etc. None of these exciting causes are alone competent to produce hay fever. A person who has no predisposition to it cannot take the disease from any one of the exciting causes. Indigestible food may superinduce sick headache in persons with a weak stomach, but the same food will not give sick headache to those who are very robust. It is most frequent in persons of nervous and nervo-bilious temperaments, and is confined to the temperate zone. Nervous patients are more benefited by a trip South than consumptive patients. It is hereditary. There is no other disease of which the hereditary character can be more distinctly proved by statistics. It is peculiar to modern civilization. It is increasing steadily as nervous diseases are increasing. The symptoms of the disease are markedly of a nervous character. The suddenness of the symptoms, the instantaneousness by which they may be cured, all point to the nervous character of hay fever.

An important element in the production of the disease is, next to predisposition, heat following cold. Where heat is steady, as in the South, hay fever and all nervous diseases are rare. In the absence of predisposition the exciting causes are powerless to produce the disease. It may come on in a mild form by exposure to heat or confined air at any time of the year. Like other nervous diseases, it acts vicariously, and is benefited by the tonic influence of mountain and sea air. The remedies which are most beneficial in hay fever are mere tonics. The plan of treatment which the Doctor proposes is, first, to prevent the disease; the patient should early in the spring begin a course of tonic treatment. It is probable that such a treatment would have the effect, with many cases, of bridging over the season, or, at least, of making the attacks milder. When the disease appears the great dependence must be on local treatment, combined with tonics. The Doctor, after naming some medicines which might be administered, said the theory that infusoria in the nasal organs was the cause of this disease could not be proved. It had been shown that infusoria were found in the nasal organs at all times, and even if they were found during the progress of the disease, no one could prove that they were the exciting cause. It was the common boast of the hay-fever army that the disease was peculiar to the intellectual classes. They rejoiced that however terribly they suffer, they are at least in good company. It was certainly true that the majority of cases were of a finely organized type. They were simply the persons who suffer from nervous diseases of all kinds. Concerning the pollen theory he would only say that it was entirely untenable.

The Doctor concluded by recommending a course of tonic treatment as a means of prevention, and if that failed, mountain air and local treatment as a means of cure.

John C. Peters, M.D., of New York, read a paper upon "The Stealthy Introduction and Spread of Infectious Diseases in Large Cities."

Influenza (the first disease mentioned in the paper), Dr. Peters said, has generally been regarded as the very type of an atmospheric affection, arising from some distemperature of the air, or from a special agent profusely developed in the skin, like ozone. But Parkes correctly concludes that influenza cannot be caused by a gas, for no gas could be spread very far or wide without extreme dilution, and utter dispersion and destruction. He also suggests that it cannot arise from any molecular matter driven through the air like the pollen or odor of plants, such as causes hay or rose catarrh. The conclusion is almost irresistible that the agent or cause of influenza cannot depend upon one primary and single origin. All the phenomena of its spread show that it must, in its transit, constantly and copiously reproduce itself, somewhat like the catarrhal poison of measles. There must be an incessant reproduction of the agent in each new place where it shows itself. This reproduction must either take place in the air or in the bodies of the sick. If it increases in the air, then some force successively changes the elements of the atmosphere, like in the formation of ozone, or else the increase is a vital one and constantly in the enormous development of some infectious substances.

To account for the mode and prolonged spread of influenza we must believe that the particles of the body pass off in myriads from each sick person, and either infect other persons in their immediate neighborhood while in a fresh and moist state, or else after they have dried up and become small and light, so that they can float through the air to greater or less distances and become revivified by breathing or swallowing in other persons. A careful examination of the history of the disease shows that the rapidity of its progress has often been exaggerated. Occasionally its advance has been very swift, yet not to such an extent as is commonly assumed, while sometimes it has even traveled slowly. It is said to have overspread Europe in six weeks, but more frequently it has required over six months. It has on some occasions taken weeks or months to spread from England to Scotland, but in 1832 needed no less than eight months to spread over Germany. Though proceeding in direct lines it does not always attack all points alike. In coming into cities it generally attacks a few families at first and then spreads rapidly. A vast amount of superficial observation has clouded over the real natural history of the disease.

The history of hay fever and dandy fever was then traced, and the Doctor continued: The West Indies may now be regarded as the focal area of yellow fever, whence it is distributed to other parts of the world, even including New Orleans, Mobile, Pensacola, and all parts of the United States. From 1674 to 1850 it had never been known in South America south of the river Amazon, doubtless because trade with the West Indies was then little carried on, but it had frequently appeared in the United States. It is permanently present in Cuba, St. Thomas and St. Domingo, doubtless maintained by the filthy habits of the natives and the heat and malaria of the climate. It has been sent from the West Indies to Barcelona, Gibraltar, Lisbon, Oporto, France, and even directly to England. It is generally communicated to ships by persons and clothing, but especially by the filthy water and mud of yellow fever ports soaking into the holds of vessels. Its infectious nature at times becomes one of its most destructive features.

Typhus, typhoid, and relapsing fever, measles, scarlet fever, whooping cough, small-pox, and cholera, were then briefly considered, and concluded the paper.

On motion, Dr. Peters' paper was referred to the Publication Committee. A paper upon "Suicide in large cities, with reference to certain sanitary conditions which tend to prevent its moral and physical causes," by Allan McLane Hamilton, M.D., of New York, was referred to the Publication Committee, without its being read.

The Association then adjourned to 3 o'clock.

AFTERNOON SESSION.

The Association re-assembled at 3 o'clock, Dr. Edwin M. Snow in the chair.

The first paper read was upon "The influence of the high altitudes and climate of the tableland country of the Rocky Mountain region upon health and disease," by B. E. Fryer, M.D., surgeon of the United States army. The Doctor states that, in connection with the subject of health, the meteorology of the whole region is of peculiar interest. The annual rainfall in the eastern portion of it will not probably average over twenty inches, and diminishes westward until the mountains are reached, near which it will not average much more than ten inches annually. Fogs are very infrequent and of short duration. The winds have considerable force more or less continuously; this is specially the condition in Western Kansas and Eastern Colorado, though it applies to the whole region. The temperature of the high altitudes is not so low as might be expected. At Fort Walker, in Kansas, at an elevation of 1856 feet above the sea, the mean temperature is 51° Fahr. The temperature of the lower levels of the eastern part of the plateau may and often does reach 105° Fahr. in summer, but the heat is rarely oppressive. This will be readily understood when remembering the small amount of moisture in the atmosphere and the consequent rapid surface evaporation. Winter, in the latitude of Kansas and Colorado, rarely commences till the middle or end of December, and spring generally appears at the end of February. Ozone is believed to exist in large quantities in the atmosphere of the plateau region, though no observations as to this were made, or were obtainable.

Among the diseases that are of rare occurrence may be included those of malarial origin, with the exception of the valleys of the streams in the lower levels of the plateau, and not often there. Ordinary scrofulous troubles are unknown, and diseases of the joints and bones almost so. Inflammation of the lungs, contrary to what is generally believed, is far from being infrequent. Over a large portion of the greater altitudes of the Rocky Mountain region and in New Mexico, and some parts of Colorado, it has several times assumed almost an epidemic character. At the lesser altitudes, both pneumonia and erysipelas are of rare occurrence, and this is especially the case so far as the former of the two diseases is concerned. Neither asthmatic difficulties nor chronic bronchial troubles are of very frequent occurrence in the older inhabitants or native people. Among new comers, if there is an asthmatic predisposition, the disease will certainly be provoked. An entirely satisfactory hypothesis for this has not been framed. It is, however, believed that some peculiar cause exists other than that which might be referred to the necessarily increased action of the lungs, dependent on the elevation.

The Doctor, alluding to the popular belief that the Rocky Mountain region is beneficial to persons suffering from pulmonary complaints, says he is convinced the belief is an error. Many cases of phthisis sent there from the East were not only not improved, but made worse. The disinclination and inability of patients to take exercise was referred to as one cause of the ill effects of the change. All consumptives should be excluded from the higher altitudes. There are, however, at lower elevations, points where there are all the advantages of dry air with day after day of sunshine. This region is found in Kansas, a large portion of Colorado and

Southern New Mexico. The paper concludes with the assertion that although a healthy individual will, with proper care, gain in vigor at high altitudes, and certain forms of debility, without organic lesions, do well, persons suffering from pulmonary diseases should not ascend to the altitudes beyond three or four thousand feet above the level of the sea.

J. S. Billings, M.D., Assistant Surgeon of the United States army, presented an abstract of special reports by army medical officers on the effect of mountain climates upon health. The conclusions drawn from the statistics gathered in the West were similar to those arrived at by Doctor Fryer in making his researches.

Dr. A. N. Bell, of Brooklyn, N. Y., then read a paper on "Perils of the Schoolroom, which demand the attention of Educational and Sanitary Authorities." The paper consisted of reports of the condition of schools in Brooklyn, New York, and other cities, showing that, with few exceptions, the pupils of public schools in almost all cities were confined in illy-ventilated rooms, and exposed to the poisonous influences of impure air, malaria from bad drainage, etc. All the papers presented were referred to the Committee on Publication. A brief conference upon "Laws and Methods of the Public Health Service of the Different Cities" was then participated in by members of the association, after which the association adjourned until evening.

### EVENING SESSION.

Hon. L. H. Steiner, M.D., of Maryland was next introduced. His discourse was upon "Health, a Prerequisite of National Success in Peace and War." His address urged the subject of health upon the National Government as of paramount importance. Every hour of sickness is so much pecuniary loss to the nation. If all this could be computed, the value of good hygienic regulations could be understood. It is a terrible period in the history of a nation when its citizens commence to disregard the regulations of bodily health. In times of peace healthy minds are requisite for the advancement of the country in the path of civilization, and in times of war for the promotion of the physical and mental strength of contending armies. A legitimate deduction is that it is incumbent upon the Government to enact laws regulating the sanitary condition of the cities and towns, and to spread such information before the people as will aid in securing the greatest possible prevention of disease.

Dr. Agnew was next introduced. While he was aware of the duties of individuals, the public, and the Government, in providing ample accommodations for the sick, he thought the management of hospitals should be so regulated that so far as consorts with the welfare of the sick, the hospital doors shall be thrown open to the medical profession.

Resolutions of thanks to Messrs. Eaton and Steiner were adopted, and the meeting adjourned.

### FOURTH DAY.

The last session of the annual meeting of the American Public Health Association began at 11 o'clock. The attendance was quite good. President Stephen Smith, M.D., occupied the chair. A number of gentlemen, whose names had been submitted to the Executive Committee, were elected to membership.

The Association then went into an election for officers. The present presiding officer, Stephen Smith, M.D., of New York, declined the compliment of a renomination, which was tendered, and the election resulted as follows:—

President, J. M. Toner, M.D., of Washington; First Vice President, E. M. Snow, M.D., of Rhode Island; Second Vice President, Professor Henry Hartshorne, of Philadelphia; Secretary, Elisha Harris, M.D., of New York; Treasurer, John H. Rauch, of Illinois. Executive Committee, J. S. Billings, United States Army; Stephen Smith, New York; Moreau Morris, New York; J. J. Woodward,

United States Army; James A. Steuart, Baltimore, and A. N. Bell, New York.

A resolution instructing the Executive Committee to enter into communication with the health boards all over the country as to quality and condition of water, and the general sanitary condition of the cities which they represent, with a view of obtaining a full knowledge on this subject, was passed, and after disposing of some unimportant matters, the Association adjourned *sine die*.

